# When is vancomycin prophylaxis necessary? Risk factors for MRSA surgical site infection

**DOI:** 10.1017/ash.2024.7

**Published:** 2024-01-25

**Authors:** Cynthia T. Nguyen, Rachel Baccile, Amanda M. Brown, Alison K. Lew, Jennifer Pisano, Natasha N. Pettit

**Affiliations:** 1 Department of Pharmacy, University of Chicago Medicine, Chicago, IL, USA; 2 The Center for Health and the Social Sciences, The University of Chicago, Chicago, IL, USA; 3 Department of Infection Prevention and Control, University of Chicago Medicine, Chicago, IL, USA; 4 Department of Medicine, Section of Infectious Diseases and Global Health, Chicago, IL, USA

## Abstract

**Background:**

The 2022 SHEA/IDSA/APIC guidance for surgical site infection (SSI) prevention recommends reserving vancomycin prophylaxis to patients who are methicillin-resistant *Staphylococcus aureus* (MRSA) colonized. Unfortunately, vancomycin prophylaxis remains common due to the overestimation of MRSA risk and the desire to cover MRSA in patients with certain healthcare-associated characteristics. To optimize vancomycin prophylaxis, we sought to identify risk factors for MRSA SSI.

**Methods:**

This was a single-center, case-control study of patients with a postoperative SSI after undergoing a National Healthcare Safety Network operative procedure over eight years. MRSA SSI cases were compared to non-MRSA SSI controls. Forty-two demographic, medical, and surgical characteristics were evaluated.

**Results:**

Of the 441 patients included, 23 developed MRSA SSIs (rate = 5.2 per 100 SSIs). In the multivariable model, we identified two independent risk factors for MRSA SSI: a history of MRSA colonization or infection (OR, 9.0 [95% CI, 1.9–29.6]) and hip or knee replacement surgery (OR, 3.8 [95% CI, 1.3–9.9]). Hemodialysis, previous hospitalization, and prolonged hospitalization prior to the procedure had no measurable association with odds of MRSA SSI.

**Conclusions:**

Patients with prior MRSA colonization or infection had 9–10 times greater odds of MRSA SSI and patients undergoing hip and knee replacement had 3–4 times greater odds of MRSA SSI. Healthcare-associated characteristics, such as previous hospitalization or hemodialysis, were not associated with MRSA SSI. Our findings support national recommendations to reserve vancomycin prophylaxis for patients who are MRSA colonized, as well as those undergoing hip and knee replacement, in the absence of routine MRSA colonization surveillance.

## Background

Surgical site infection (SSI) is the most common reason for unplanned post-procedure admissions in the United States^1^ and presents an increasing public health challenge due to significant morbidity and mortality among patients, higher costs, and decreased reimbursement for health systems.^2,3^ Between 2015 and 2017, *Staphylococcus aureus* accounted for 18% of SSI cases reported to the National Healthcare Safety Network (NHSN), with 53% of these due to methicillin-resistant *S. aureus* (MRSA).^4^ Methicillin-resistant *Staphylococcus aureus* SSIs have been associated with greater mortality, longer length of stay, and higher hospital costs compared to SSIs caused by other organisms.^5^ As such, providers are particularly sensitive to the risk of MRSA SSIs.

Vancomycin is commonly administered preoperatively to prevent MRSA SSI. However, much of this is discordant with updated guideline recommendations and is unnecessary.^6^ The prior 2013 ASHP/IDSA/SIS/SHEA Clinical Practice Guideline for Antimicrobial Prophylaxis in Surgery recommended vancomycin for patients with known MRSA colonization and for patients at high risk for MRSA colonization, in the absence of surveillance data. Examples included are patients with recent hospitalization, nursing home residents, and hemodialysis patients.^7^ As many hospitals lack locally validated risk factors, many hospitals have relied on these healthcare-associated characteristics to guide vancomycin prophylaxis. In 2022, SHEA/IDSA/APIC updated their recommendations to state that vancomycin prophylaxis should only be considered in patients known to be MRSA colonized or in the setting of a proven MRSA SSI outbreak.^8^ Supporting data for this change include studies demonstrating no difference in SSI prevention among patients who received MRSA-active prophylaxis compared to no MRSA-active prophylaxis and the toxicities associated with vancomycin prophylaxis.^9–11^ However, specific data evaluating MRSA SSI risk factors remain limited and the removal of these healthcare-associated characteristics from the recommendation is not specifically addressed.

Reliance on general healthcare-associated characteristics has resulted in an overestimation of MRSA risk and subsequent vancomycin overuse in the surgical setting. Vancomycin overuse has profound implications, including potential surgical delays and acute kidney injury.^6^ To optimize vancomycin use in this setting, a better understanding of risk factors for MRSA SSI is needed. The goal of this study was to identify risk factors for MRSA SSI compared to non-MRSA SSI among patients undergoing an NHSN operative procedure in order to provide more specific guidance for vancomycin prophylaxis.

## Methods

### Study population and data collection

Patients who were diagnosed with a postoperative surgical site infection after undergoing a clean or clean-contaminated NHSN operative procedure between July 1, 2014 and August 30, 2022 were included.^9^ Patients less than 18 years old, those receiving antibiotics for the treatment of active infection prior to surgery, and those with surgical wound class III–IV were excluded.

Patients undergoing NHSN operative procedures were identified using the Epic® Bugsy SSI application with keywords that denote infection. These patients were reviewed by an Infection Preventionist and determined if the case met NHSN SSI criteria. Data on demographic characteristics, medical, and surgical details, including previous MRSA colonization or infection within the past five years, were obtained through retrospective evaluation of the electronic health record. Healthcare-associated characteristics were also collected and were defined as: being hospitalized for longer than 72 hours within 90 days prior to the procedure; current hospitalization for 72 hours prior to the inpatient procedure; skilled nursing facility, long-term acute care, or nursing home resident; receiving hemodialysis; and receiving home wound care 30 days prior to the procedure. Available outside hospital data were reviewed through the health information exchange (Epic® Care Everywhere). Surgical site infection depth was defined using NHSN criteria.^12^ *Clostridioides difficile* (*C. difficile*) infection was defined as the presence of diarrhea, a positive test for *C. difficile*, and the receipt of treatment for *C. difficile* infection.

### Setting

This study was conducted at the University of Chicago Medicine, an 811-bed academic and trauma center in Chicago, Illinois, and received a formal Determination of Quality Improvement status according to institutional policy. As such, this initiative was deemed not human subjects research and was therefore not reviewed by the Institutional Review Board.

Throughout the study period, institutional antimicrobial prophylaxis guidance based on procedure type was posted on the hospital website and integrated into preoperative order sets. The addition of vancomycin prophylaxis was recommended in patients who have a history of MRSA infection or colonization; hemodialysis; recent hospitalization for longer than 72 hours in the prior 90 days; for patients who undergo surgery after 72 hours of hospitalization; and any surgery involving placement of prosthetic material (e.g., hip and knee replacement surgeries). Additionally, for patients with a severe beta-lactam allergy who cannot receive preferred therapy, vancomycin was recommended (usually in combination with another antibiotic). Adherence to institutional antibiotic prophylaxis recommendations was not monitored or tracked during the study period and prescribers were permitted to deviate from institutional guidance.

Throughout the study period, there was no routine preoperative MRSA colonization screening. Routine staphylococcal decolonization (e.g., intranasal iodine or mupirocin) was recommended prior to orthopedic and cardiac procedures. Chlorohexidine skin treatments were recommended the night before and morning of surgery for all procedures with an incision. Oral antimicrobial prophylaxis and mechanical bowel preparation were also recommended prior to elective colorectal surgery. Adherence to institutional recommendations was not monitored or tracked during the study period and providers were permitted to deviate from institutional guidance.

### Study design

A case-control study was performed to identify risk factors for developing an MRSA SSI compared to any other SSI. Cases were defined as patients for whom results of the surgical site wound culture found MRSA (MRSA SSI). Controls were defined as patients who had a surgical site infection and the wound culture did not grow MRSA (non-MRSA SSI), which included patients who did not have cultures obtained or had no growth on culture.

### Sample size and minimum detectable association

Published literature suggests that patients with a positive nasal MRSA polymerase chain reaction (PCR) screen have 9-fold greater odds of developing a subsequent MRSA SSI compared to those with a negative MRSA PCR screen.^13^ Given that *S. aureus* was present in 15.4% of SSIs reported to NHSN and 39.2% of those tested were MRSA, we expected approximately 7% of all SSIs in our sample to be MRSA.^14^ Based on an odds ratio of 9, a prevalence of 7%, a baseline prevalence of MRSA colonization of 2%,^15^ and confidence level of 95%, 24 patients with MRSA SSI will be needed to achieve a desired power of 80%.

Power calculations were also conducted based on our sample (Table 1) to estimate the minimum detectable association. We were 80% powered to detect odds ratios (ORs) between 3.7 and 9.0, assuming non-MRSA cohort means between 2% and 30%, an upward association, an α level of 0.05, and a two-tailed test (Supplemental Figure 1).

**Table 1.** Patient and procedure characteristics

	Patients with non-MRSA SSI	Patients with MRSA SSI	*p*-value
	*N* = 418	*N* = 23	
**Demographics**			
Age, median [IQR], and years	55 [40, 66]	53 [43, 69]	0.558
Male	120 (28.7)	10 (43.5)	0.130
Race			
White	184 (44.0)	6 (26.1)	0.086
Black	195 (46.7)	12 (52.2)	
Other race or unknown	14 (3.3)	0 (0.0)	
Ethnicity			
Hispanic	26 (6.2)	4 (17.4)	0.048
Not Hispanic or unknown	392 (92.8)	19 (73.9)	
Beta-lactam allergy	89 (21.3)	1 (4.3)	0.044
History of MRSA colonization or infection	9 (2.2)	4 (17.4)	0.001
**Comorbidities**			
Diabetes	104 (24.9)	8 (34.8)	0.268
Active or previous smoker	192 (45.9)	11 (47.8)	0.850
Hypertension	221 (52.9)	9 (39.1)	0.205
Asthma or COPD	87 (20.8)	5 (21.7)	0.205
BMI, median [IQR]	31 [26, 37]	32 [27, 41]	0.180
BMI > 30	227 (54.3)	14 (60.9)	0.556
Immunocompromised	132 (31.6)	4 (17.4)	0.166
Malignancy	118 (28.2)	2 (8.7)	0.037
Receiving prednisone 10mg > = 30 days	11 (2.6)	1 (4.3)	0.384
HIV	2 (0.5)	1 (4.3)	0.051
Primary immunodeficiency	1 (0.2)	0 (0.0)	0.359
History of solid organ transplantation	1 (0.2)	0 (0.0)	0.359
**Procedure characteristics**			
Index procedure			
Hip or knee replacement	36 (8.6)	7 (30.4)	0.003
Colorectal surgery	125 (29.9)	3 (13.0)	0.086
Abdominal or vaginal hysterectomy	81 (19.4)	3 (13.0)	0.543
C-section	58 (13.9)	3 (13.0)	0.932
CABG or open chest procedure	54 (12.9)	2 (8.7)	0.705
Spinal fusion or laminectomy	24 (5.7)	2 (8.7)	0.403
Gastric surgery	13 (3.1)	2 (8.7)	0.126
Vascular surgery	22 (5.3)	1 (4.3)	0.856
Esophageal surgery	5 (1.2)	0 (0.0)	0.767
Emergent procedure	17 (4.1)	0 (0.0)	0.580
Wound class			
I	141 (33.7)	12 (52.2)	0.074
II	277 (66.3)	11 (47.8)	
Procedure length, median [IQR], and hours	3.9 [2.3, 5.5]	2.6 [2.1, 4.7]	0.289
ASA status			
1	5 (1.2)	0 (0.0)	0.880
2	116 (27.8)	6 (26.1)	
3	235 (56.2)	15 (65.2)	
4	62 (14.8)	2 (8.7)	
Received preoperative vancomycin	102 (24.4)	9 (39.1)	0.181
Hours before incision vancomycin infusion started, median [IQR]	1.2 [0.5, 1.7]	1.6 [1.2, 1.8]	0.670
Received other preoperative antibiotics	402 (96.2)	21 (91.3)	0.190
Received systemic antibiotics in the 90 days prior to surgery	144 (34.4)	8 (34.8)	0.926
Received MRSA decolonization prior to surgery	36 (8.6)	4 (17.4)	0.139
Received antibiotic bowel preparation/decontamination prior to surgery	68 (16.3)	2 (8.7)	0.417
**Healthcare-associated characteristics**	126 (30.1)	6 (26.1)	0.857
Hospitalized for >72 hrs within 90 days prior to procedure	77 (18.4)	4 (17.4)	0.975
Hospitalized for >72 hrs immediately prior to inpatient procedure	69 (16.5)	2 (8.7)	0.401
Received home wound care 30 days prior to procedure	21 (5.0)	2 (8.7)	0.317
SNF, LTAC, or nursing home resident immediately prior to procedure	14 (3.3)	0 (0.0)	0.698
CKD on chronic hemodialysis	10 (2.4)	0 (0.0)	0.894

Note: Data are counted (%) unless otherwise specified. ASA, American Society of Anesthesiologists; SNF, skilled nursing facility; LTAC, long-term acute care.

### Statistical analysis

Identification of potential risk factors for MRSA SSI was initially determined by univariate analysis. We used unconditional logistic regression with Firth’s penalized likelihood to estimate crude and adjusted ORs and 95% confidence intervals (CIs) for the association between risk factors and MRSA SSI risk. We used Firth’s penalized likelihood to reduce bias in estimates with small sample sizes.^16^ Variables associated with MRSA SSI with a *P* value <0.1 in univariate analysis were candidates for multivariate modeling. The final model was selected using backward stepwise logistic regression. Goodness of fit of the final model was assessed using the area under the curve (AUC). A *P* value of <0.05 was defined as statistically significant.

To account for potential confounding from receipt of vancomycin prophylaxis among patients perceived as high risk for MRSA SSI, we performed a sensitivity analysis. Using the Woolf test, we tested for homogeneity of ORs stratified by receipt of prophylaxis for all factors included in the institutional antimicrobial prophylaxis guidance (described above). Additionally, we calculated ORs for these factors in the subset of patients who did not receive prophylaxis. All analysis was conducted in R version 4.2.2^17^ using the logistf package version 1.25.0.^18^

## Results

### Patient population

During the study period, 550 SSIs were identified and 109 patients were excluded, leaving 441 patients in the analysis. Fifty-three patients were excluded due to receipt of antibiotics for the treatment of active infection prior to surgery, 53 patients underwent a procedure with surgical wound class III-IV, and three patients were less than 18 years old. Surgical site infections were most commonly identified after a colorectal surgery (*n* = 128, 29%) or hysterectomy (*n* = 84, 20%) (Table 1).

### Antibiotic prophylaxis

Of the 441 patients included in the cohort, 423 (96%) received preoperative antibiotic prophylaxis. The most common antibiotics received were cefazolin (*n* = 180, 43%), cefoxitin (*n* = 158, 37%), and ampicillin (*n* = 156, 37%). Consistent with institutional guidelines, ampicillin was usually given with cefoxitin to cover *Enterococcus* spp. prior to colorectal procedures. One-hundred and eleven (26%) patients received vancomycin prophylaxis either with (*n* = 101, 91%) or without (*n* = 10, 9%) another antibiotic. Among the 13 patients with previous MRSA colonization or infection, 9 (69%) received vancomycin. Among the 132 patients with a healthcare-associated characteristic, 46 (35%) received vancomycin.

### Prevalence of MRSA SSI among all SSIs

Wound cultures were obtained for 360 (82%) patients. In total, 46.1% (*n* = 166) of wound cultures were polymicrobial and 44.7% (*n* = 161) monomicrobial. Thirty-three patients had cultures where either no organism (*n* = 16, 4.4%) or no predominant organism was identified (*n* = 17, 4.7%). Of the 441 patients included in the cohort, 23 developed MRSA SSIs, a rate of 5.2 per 100 SSIs. The most common organisms among the non-MRSA SSI cohort were *Enterococcus faecalis* (*n* = 80, 19.1%), coagulase-negative staphylococci (*n* = 68, 16.3%), methicillin-susceptible *Staphylococcus aureus* (MSSA) (*n* = 54, 12.9%), *E. coli* (*n* = 45, 10.8%), *Pseudomonas* spp. (*n* = 43, 10.3%), and *Streptococcus* spp. (*n* = 37, 8.9%). In the MRSA cohort, 6 patients (26.1%) had more than one organism found, the most common of which was *Pseudomonas* spp. (*n* = 3, 13.0%).

### MRSA SSI risk analysis

Table 1 summarizes the characteristics of the cases and controls. By univariate logistic regression analysis, we found that three covariates were associated with greater odds of MRSA SSI: Hispanic ethnicity, hip or knee replacement surgery, and having a history of MRSA colonization or infection. We found two covariates were associated with lower odds of MRSA SSI: beta-lactam allergy, and having any malignancy (Table 2).

**Table 2.** Crude and adjusted odds ratios of characteristics associated with methicillin-resistant *Staphylococcus aureus* surgical site infection (MRSA SSI)

	Univariate		Multivariate	
	OR	95 % CI	aOR	95 % CI
Hip or knee replacement	4.76	(1.79, 11.72)	4.13	(1.45, 10.82)
History of MRSA colonization or infection	9.95	(2.71, 32.19)	9.09	(2.19, 33.11)
Malignancy	0.29	(0.06, 0.94)	0.35	(0.07, 1.14)
Beta-lactam allergy	0.25	(0.03, 0.97)	–	–
Hispanic	3.42	(1.01, 9.54)	3.64	(0.94, 11.48)

Note: Beta-lactam allergy was excluded from the multivariate model after backward stepwise regression.

With regard to healthcare-associated characteristics, hemodialysis (OR, 0.8 [95% CI, 0.0–6.8]), hospitalization for greater than 72 hours in the past 90 days (OR, 1.0 [95% CI, 0.3–2.7]), hospitalization greater than 72 hours prior to the procedure (OR, 0.6 [95% CI, 0.1–1.9]), being a long-term care resident (OR, 0.6 [95% CI, 0.0–4.7]), and home wound care (OR, 2.1 [95% CI, 0.4–7.3]) had no measurable association on odds of MRSA SSI. Receipt of preoperative prophylaxis vancomycin (OR, 2.0 [95% CI, 0.8–4.7]) also had no measurable association with odds of MRSA SSI.

In the multivariable model (area under the receiver operating characteristic curve [AUC], 0.73), we identified two independent risk factors for MRSA SSI: receiving hip or knee replacement surgery and a history of MRSA colonization or infection (Table 2). Of the 23 cases with an MRSA SSI, 9 (39.1%) had at least one of the independent risk factors identified in the multivariate analysis (hip or knee replacement surgery or a history of MRSA colonization), compared to 45 (10.8%) of the control non-MRSA SSI cohort (OR, 5.4 [95% CI, 2.2–12.8]).

### Sensitivity analyses

Woolf tests of homogeneity indicated that there was no evidence of differing ORs stratified by receipt of preoperative vancomycin for the recommended risk factors (history of MRSA colonization, *p* = 0.86; hip or knee replacement surgery, *p* = 0.74; history of prior hospitalization in past 90 days, *p* = 0.95; current hospitalization of >72 hours, *p* = 0.99; on hemodialysis, *p* = 0.48). This indicates that among our sample, receipt of prophylactic vancomycin did not modify the risk of MRSA SSI in the overall population, nor in relation to the recommended risk factors.

Additionally, in analysis of the subset of patients that did not receive prophylaxis vancomycin (*n* = 330; MRSA *n* = 14, non-MRSA *n* = 316), we again found no measurable association on odds of MRSA SSI among those with any healthcare-associated characteristics (Supplemental Table 1). Odds ratios for history of MRSA colonization and receiving a hip or knee replacement were similar among the overall population, however, CIs were wider, reflecting the smaller sample size.

When patients with no culture data were excluded, the results of the multivariable model were similar. A history of MRSA colonization or infection (OR, 9.87 [95% CI, 2.23–39.99]) and hip or knee replacement surgery (OR, 3.44 [95% CI, 1.19–9.20]) were both associated with MRSA SSI and there was no measurable association on odds of MRSA SSI among those with any healthcare-associated characteristics (Supplemental Table 2).

### Clinical outcomes

In the study population, there were no statistically significant differences in mortality, readmission, or postoperative *C. difficile* rates among patients with and without MRSA SSIs (Table 3). Patients with MRSA SSI had a longer time to SSI development (median 20 days vs 15 days, *p* = 0.04).

**Table 3.** Clinical outcomes of patients with non-MRSA SSI and MRSA SSI

	Patients with non-MRSA SSI	Patients with MRSA SSI	*p*-value
	*N* = 418	*N* = 23	
Days to SSI (median)	15 [10, 22]	20 [16, 23.5]	0.04
SSI depth			
Superficial incisional	232 (55.5)	12 (52.2)	0.17
Deep incisional	54 (12.9)	6 (26.1)	
Abscess or organ space	132 (31.6)	5 (21.7)	
Length of stay for procedure, days (median)	7 [4, 14]	5 [3, 9.5]	0.12
Mortality within 90 days after the procedure	11 (2.6)	0 (0.0)	>0.99
Infection-related readmission within 90 days after the procedure	225 (53.8)	12 (52.2)	>0.99
Postoperative *C. difficile* infection within 30 days after the procedure	9 (2.2)	0 (0.0)	>0.99

Note: SSI, surgical site infection, MRSA, methicillin-resistant *Staphylococcus aureus*.

## Discussion

We examined the relationship between 42 characteristics and MRSA SSI in 441 patients with an SSI. Consistent with prior studies, we found the likelihood of MRSA SSI to be approximately 3–4 times higher among patients undergoing hip or knee replacement and 9–10 times higher among patients with a history of MRSA colonization.^4,13^ Importantly, we did not find evidence of a relationship between health-care-associated characteristics and MRSA SSI, including current hospitalization of >72 hours, hemodialysis, and hospitalization for longer than 72 hours in the past 90 days. These findings support the 2022 SHEA/IDSA/APIC updated recommendation to reserve vancomycin prophylaxis for patients who are colonized with MRSA.

Routine preoperative screening for MRSA colonization presents challenges and is not often possible. Consequently, many hospitals rely on previously identified MRSA colonization risk factors, such as recent hospitalization, to guide vancomycin prophylaxis.^7^ In recent years, these risk factors have been called into question for many disease states, such as community-acquired pneumonia, as they are poorly predictive of MRSA infection.^19^ Updated SSI prevention guidance^8^ does not recommend the use of these healthcare-associated characteristics. Still, dogma surrounding these patient characteristics has contributed to the increased perception of MRSA risk and contributed to vancomycin overuse.^6^

Vancomycin overuse in the surgical setting presents several logistical and patient safety concerns. Since vancomycin is weight-based and requires a prolonged infusion time prior to incision, there are several challenges to preoperative administration. Time needed for medication preparation, delivery, storage, and administration can complicate preoperative care and lead to procedure delays. Vancomycin use is associated with postoperative acute kidney injury and other adverse events.^6,20^ Antibiotics are the most commonly reported causes of perioperative anaphylaxis in the United States and the United Kingdom, driven by beta-lactams and glycopeptides (vancomycin and teicoplanin).^21^ While reactions to beta-lactam antibiotics are the most commonly reported, a prospective United Kingdom registry of 286 cases found that although teicoplanin comprised only 12% of antibiotic exposures, it caused 38% of antibiotic-induced anaphylaxis cases. The overall incidence of teicoplanin-induced anaphylaxis was 16.4 per 100,000 administrations.^22^ Reducing unnecessary glycopeptide use can limit unnecessary toxicities.

Our findings support the updated 2022 SHEA/IDSA/APIC guideline recommendations to reserve vancomycin prophylaxis for patients who are known to be MRSA colonized.^8^ We also found patients undergoing hip and knee replacement to be at higher risk of MRSA SSI, which is consistent with prior data suggesting patients undergoing procedures involving prosthetic material are at higher risk of MRSA SSI.^4^ Ideally, these patients would undergo screening for MRSA colonization prior to the procedure to identify those most likely to benefit from vancomycin prophylaxis. Using a clinical history of MRSA infection or colonization alone may be insufficient to identify preoperative MRSA colonization. Although a clinical history of MRSA infection or colonization is highly specific for MRSA colonization, Strymish and colleagues found that relying on an MRSA culture history alone may miss more than 80% of patients who are MRSA colonized by preoperative nasal PCR.^23^ If routine preoperative screening is not feasible, it may be reasonable to provide vancomycin prophylaxis to patients undergoing these procedures, in regions with higher rates of MRSA. Although this practice may still unnecessarily expose patients who are not MRSA colonized to the unnecessary toxicities described above.

Our study had several important limitations. First, as with all observational studies, we cannot infer causality from our results. Second, our small sample of 23 patients with MRSA SSI limited our power to detect ORs below 3.7 to 9, depending on the prevalence of the characteristic within the population (Supplemental Figure 1). Third, as a retrospective study, we were limited to the data available in the electronic health record. Due to lack of consistent documentation, we were unable to capture other important factors that may influence SSI risk (e.g., skin preparation). We also assumed if there was no culture data available, the patient had a non-MRSA SSI. However, when these patients were excluded, the results were similar (Supplemental Table 2). Additionally, only in the last five years, has there been increased clinical use of the MRSA nasal swab in the inpatient setting for potential vancomycin de-escalation. Consequently, most patients included in the study did not receive MRSA colonization screening and were assumed to not be colonized. Fourth, adherence to institutional guidelines for antibiotic prophylaxis was not captured and receipt of vancomycin prophylaxis might have confounded our results. However, despite institutional recommendations to use vancomycin prophylaxis in patients with healthcare-associated characteristics, only 35% of patients with these characteristics received vancomycin prophylaxis. Therefore, it is unlikely that receipt of vancomycin significantly influenced our findings. Furthermore, our sensitivity analysis comparing patients who received and did not receive vancomycin prophylaxis demonstrated that receipt of vancomycin prophylaxis did not modify the risk of MRSA SSI in our study population. Lastly, this was a single-center study and caution should be taken when extrapolating results, particularly when patient populations, infection prevention precautions, and surgical techniques may differ. This may be reflected in our low incidence of MRSA SSIs (23 cases over 8 years).

## Conclusions

Our single-center evaluation of 441 patients with SSI found that patients with a history of MRSA colonization or infection had 9–10 times greater odds of MRSA SSI, and patients undergoing hip and knee replacement had 3–4 times greater odds of MRSA SSI. Healthcare-associated characteristics, such as recent hospitalization or hemodialysis, were not associated with MRSA SSI. Despite institutional recommendations to give vancomycin prophylaxis to patients with healthcare-associated characteristics, we did not find a relationship between vancomycin administration and MRSA SSI risk. Additional research evaluating the role and cost-effectiveness of routine MRSA colonization screening to guide vancomycin prophylaxis is necessary. Our findings support guideline recommendations to reserve vancomycin prophylaxis for patients who are MRSA colonized and patients undergoing hip and knee replacement procedures.

## Supporting information

Nguyen et al. supplementary material Nguyen et al. supplementary material

## Supplementary material

For supplementary material accompanying this paper visit http://doi.org/10.1017/ash.2024.7. click here to view supplementary material
