# Supplementary material for: When is vancomycin prophylaxis necessary? Risk factors for MRSA surgical site infection
Source: Antimicrob Steward Healthc Epidemiol. 2024 Jan 25;4(1):e10. doi: 10.1017/ash.2024.7 (PMC10897724; doi:10.1017/ash.2024.7)
Supplement: Nguyen et al. supplementary material [file S2732494X2400007Xsup001.docx]

**Supplemental Figure 1. Minimum detectable effect size based on non-MRSA SSI cohort prevalence**


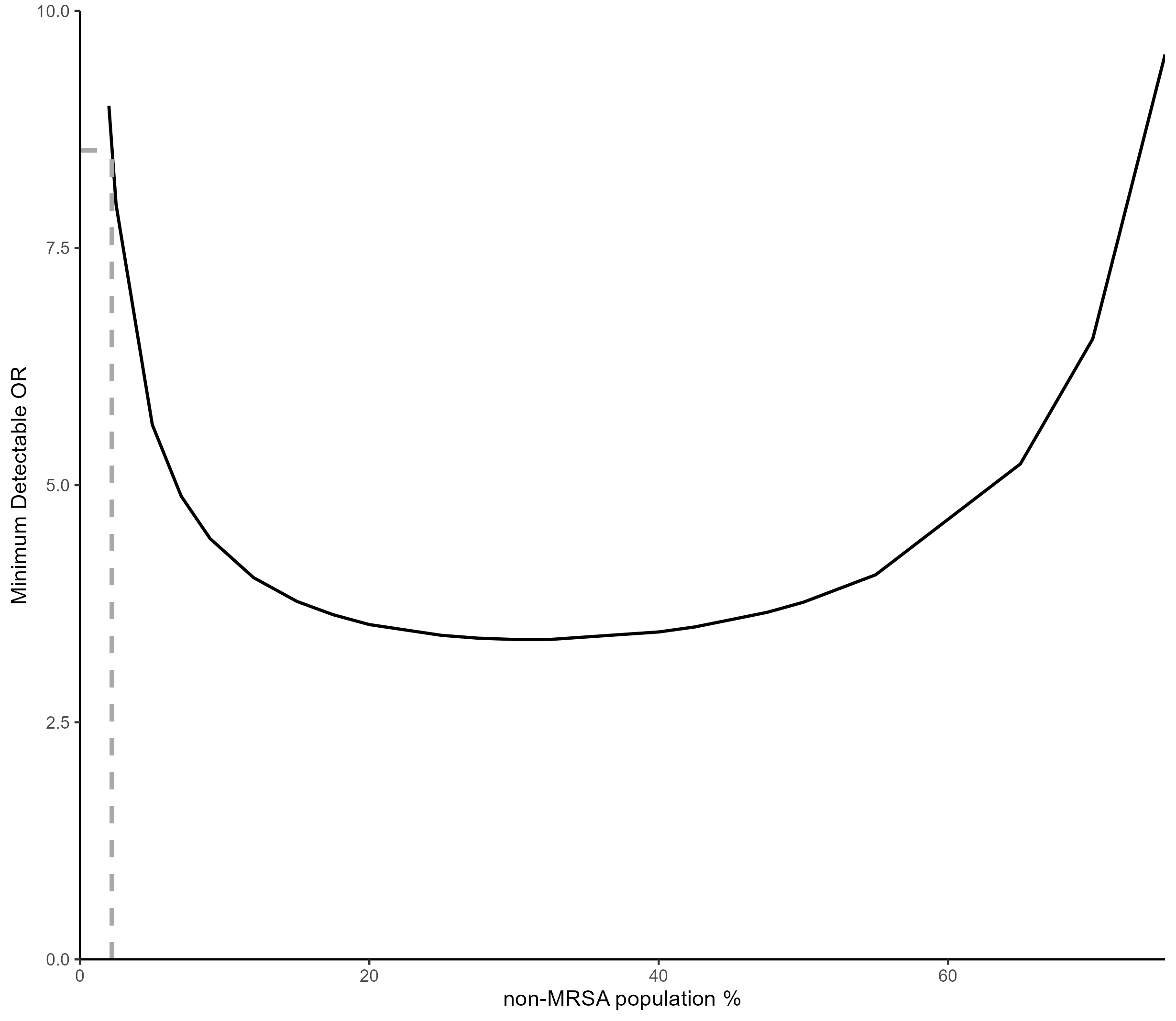


This figure was used to determine the minimum detectable effect size (y-axis) for any characteristic using the prevalence of the characteristic in the non-MRSA SSI population (x-axis). The solid curved line represents the result of the 80% power calculation. For example, in our cohort, 2.2% of the non-MRSA population had a history of MRSA colonization or infection. Therefore, our study was powered to detect a minimum OR of 8.53 (dashed line).

**Supplemental Table 1. Odds of MRSA SSI among patients with a characteristic for which vancomycin prophylaxis was recommended based on institutional guidelines, all patients and those who did not receive vancomycin**

|  | **All Patients** | | **Patients Who Did Not Receive Vancomycin** | |
| --- | --- | --- | --- | --- |
|  | **OR** | **95 % CI** | **OR** | **95 % CI** |
| Hip or knee replacement | 4.8 | (1.8, 11.7) | 6.3 | (0.6, 34.8) |
| History of MRSA colonization or infection | 9.9 | (2.1, 32.2) | 10.0 | (0.9, 65.8) |
| CKD on chronic hemodialysis | 0.8 | (0.0, 6.8) | 3.1 | (0.0, 34.0) |
| Hospitalized for 72 hrs within 90 days prior to procedure | 1.0 | (0.3, 2.7) | 1.0 | (0.2, 3.6) |
| Hospitalized for >72 hrs immediately prior to inpatient procedure | 0.6 | (0.1, 1.9) | 0.6 | (0.1, 2.7) |
| SNF, LTAC, or nursing home resident immediately prior to procedure | 0.6 | (0.0, 4.7) | 1.3 | (0.0, 10.9) |
| Received home wound care 30 days prior to procedure | 2.1 | (0.4, 7.3) | 2.3 | (0.2, 10.6) |

**Supplemental Table 2. Crude and adjusted odds ratios of characteristics associated with MRSA SSI, excluding patients who did not have an SSI culture obtained**

|  | Univariate | | Multivariate | |
| --- | --- | --- | --- | --- |
|  | OR | 95 % CI | aOR | 95 % CI |
| Hip or knee replacement | 3.87 | (1.45, 9.56) | 3.44 | (1.19, 9.20) |
| History of MRSA colonization or infection | 11.76 | (3.04, 42.35) | 9.87 | (2.23, 39.99) |
| Malignancy | 0.27 | (0.05, 0.85) | 0.31 | (0.06, 1.03) |
| Beta-lactam allergy | 0.24 | (0.03, 0.95) | – | – |
| Hispanic | 3.40 | (0.99, 9.68) | 4.19 | (1.06, 13.83) |

Note: Beta-lactam allergy was excluded from the multivariate model after backward stepwise regression
